# Phytoglobin Scavenging of Nitric Oxide Is Associated With Ethylene Reduction and Drought Tolerance in Oat (*Avena sativa*)

**DOI:** 10.1111/ppl.70597

**Published:** 2025-10-26

**Authors:** Gracia Montilla‐Bascon, Simona M. Cristescu, Luis A. J. Mur, Elena Prats

**Affiliations:** ^1^ CSIC Institute for Sustainable Agriculture Córdoba Spain; ^2^ Radboud University Nijmegen Nijmegen the Netherlands; ^3^ Department of Life Science Aberystwyth University Aberystwyth UK

**Keywords:** drought tolerance, ethylene, nitric oxide, oat

## Abstract

Drought stress significantly impacts crop productivity and plant physiology. Nitric oxide (NO) signalling is essential for drought tolerance. This study explores the relationship between in vivo NO levels, mediated by NO scavenging phytoglobin (encoded by *Pgb*, non‐symbiosis associated hemoglobin), and drought tolerance in oat ( *Avena sativa* ). Real‐time in vivo NO measurements suggested increased production under moderate to high water stress in the susceptible cultivar Flega compared to the resistant Patones. This elevated NO correlated with increased senescence in Flega. Conversely, the resistant cultivar Patones showed a marked increase in *Pgb* gene expression, which correlated with reduced NO levels in vivo. This suggested that *Pgb* acts as a protective mechanism against NO‐induced stress. Water stress‐induced NO increases fed into the polyamine pathway, leading to a significant rise in arginine decarboxylase (*ADC*) expression, leading to putrescine accumulation in the susceptible cultivar, whereas the resistant Patones maintained lower *ADC* expression and polyamine levels. Elevated in vivo ethylene production was also observed in the susceptible cultivar Flega, correlating with severe drought‐induced senescence symptoms and linked to the naturally high NO levels in this cultivar. Assessment of other oat genotypes confirmed a negative correlation between *Pgb* expression and drought symptoms. These results underscore an important role of phytoglobins in modulating NO levels to counter drought in oat and suggest a potential target for genetic improvement of oat for drought tolerance.

## 1 Introduction

Oat ( *Avena sativa* L.) is a cereal crop that, globally, is grown on more than 10 million hectares (FAO 2023). In the Mediterranean rim, the area of oat cultivation has increased at a rate of 7500 ha/year over the last years. This is due to the ability of oat to grow on a wide range of soil types and perform better than other small‐grain cereals on marginal soils (Stevens et al. 2004). However, oat is particularly sensitive to drought due to an elevated transpiration rate, leading to higher water requirements than most other small‐grain cereals (Ehlers 1989). Drought and heat result in grain abortion and the formation of empty spikelets (Canales, Rispail, Garcia‐Tejera, et al. 2021; Sanchez‐Martin et al. 2017).

Understanding how plants respond to drought remains a central challenge in plant science. Drought induces a myriad of changes in the plants at gene, protein, and metabolite levels (reviewed in Ilyas et al. 2021). Within all these cellular changes, plant signals function as regulatory nodes linking and reprogramming the complex stress adaptive genetic/metabolic pathways (Golldack et al. 2014; Canales, Montilla‐Bascón, Gallego‐Sánchez, et al. 2021). Amongst these signals, nitric oxide (NO) has emerged as an important player in plant responses to drought (Hebelstrup et al. 2014; Montilla‐Bascon et al. 2017). NO affects various physiological parameters like carbon dioxide assimilation rate, transpiration rate, and stomatal conductance. Initially considered as an air pollutant with inhibitory effects on plant growth, including reduced photosynthesis in oat and alfalfa (Hill and Bennett 1970), NO has since been shown to have both inhibitory and stimulatory effects depending on concentration, timing, and context (Zhao et al. 2020; Batista et al. 2018; De Sousa et al. 2020; Zangani et al. 2018).

NO also influences gene expression related to stress adaptation, affecting processes such as antioxidative defense, photosynthesis, hormonal signaling, and metabolism. NO has also been proposed to crosstalk with other hormones, including abscisic acid (ABA), jasmonic acid, salicylic acid, and cytokinins, as crucial players for drought stress adaptation. Furthermore, NO also acts synergistically with signaling molecules such as hydrogen sulfide (H_2_S), hydrogen peroxide (H_2_O_2_), calcium (Ca^2+^), melatonin, polyamines, and ethylene (Mishra et al. 2021; Singhal et al. 2021; Zhang et al. 2021).

Focusing on drought, NO‐induced stomatal closure following exogenous application of a NO donor has been associated with increased tolerance to rapid dehydration in wheat seedlings (García‐Mata and Lamattina 2009). However, the role of NO in stomatal movement is complex, with some studies reporting that NO is dispensable for stomatal closure under rapid dehydration (Ribeiro et al. 2009) or even promotes stomatal opening (Batista et al. 2018; Pissolato et al. 2020; Sahay et al. 2019). This ambiguity extends to NO interactions with reactive oxygen species (ROS) and with the important drought‐responsive hormone, ABA. While NO donors can reduce ROS levels and mitigate drought symptoms (Batista et al. 2018; Farouk and AL‐Huqail 2020; Rigui et al. 2019), NO can also promote H_2_O_2_ accumulation (Shi et al. 2014). Similarly, different observations have been made on NO and ABA interactions during drought. ABA has been shown to induce NO production and stomatal closure (Neill et al. 2002), and exogenous application of NO donors induces stomatal closure in *Vicia faba* (García‐Mata and Lamattina 2009). However, NO accumulation in guard cells can also inhibit ABA‐induced closure (Casaretto et al. 2021). These contrasting observations may reflect differences in stress severity and duration, plant developmental stage, or experimental approach. Notably, most studies assessing NO effects during drought stress rely on exogenous NO donors such as sodium nitroprusside (SNP) or S‐nitrosoglutathione (GSNO), while the patterns of in vivo NO generation in intact plants undergoing water stress remain largely unexplored.

The effect of NO on plant metabolism is concentration‐dependent (Hebelstrup et al. 2006; Leshem and Haramaty 1996), reflecting the rate of its biosynthesis, displacement, and catabolism (Romero‐Puertas et al. 2004). In aerobic conditions, NO is primarily produced via nitrate reductase (NR)‐dependent reduction of nitrite, and its levels are modulated by mitochondrial processes involving NO‐associated protein 1 (NOA1), which indirectly influences NO accumulation through its role in ribosome assembly and mitochondrial function (Moreau et al. 2010). NO can also be removed from the cell via oxidation to NO_3_ through the phytoglobin–NO cycle, involving the formation of methemoglobin, an oxidized form of hemoglobin that may be converted again in the reduced form by the action of a monodehydroascorbate reductase (Igamberdiev et al. 2011). This cycle plays a key role in maintaining NO homeostasis and linking NO detoxification with energy and oxygen signalling. Among the three classes of plant hemoglobins, class 1 hemoglobins (now termed phytoglobins) have been shown to modulate NO‐mediated stress responses, including flooding and hypoxia in Arabidopsis (Hebelstrup et al. 2012), barley (Hebelstrup et al. 2014), and pathogen responses (Mur et al. 2012). In barley, transgenic overexpression of a class 1 phytoglobin (*HvPgb*1), which scavenges NO, led to reduced levels of NO and enhanced drought tolerance, accompanied by important changes in polyamine metabolism and ethylene production (Montilla‐Bascon et al. 2017). This raises the question of whether such modulation of NO levels via phytoglobins represents a naturally occurring mechanism of drought adaptation in other cereals, such as oat, or whether it is merely an artifact of genetic manipulation. Several genes coding for enzymes known to mitigate drought stress or involved in signaling and regulatory pathways have been transferred into crops through genetic engineering (Gerszberg and Hnatuszko‐Konka 2017; Krishna et al. 2019; Shinwari et al. 2020). However, despite a large number of genes introduced and expressed, their success in conferring drought tolerance in the field has been poor (Gupta et al. 2011). A recent large‐scale evaluation of over 1600 candidate genes found that only one consistently improved yield across environments, and only modestly (Khaipho‐Burch et al. 2023). The limited success may, in part, reflect the activation of pathways that are not central to the plant's native drought resistance strategy. Thus, identifying genetic determinants that play a genuine role in natural stress tolerance, those whose variation leads to meaningful phenotypic changes, remains a critical step toward improving crop resilience.

Therefore, we investigate whether the modulation of in vivo NO levels by phytoglobins is part of the natural plant strategy to cope with drought. By analyzing NO dynamics, phytoglobin expression, and associated metabolic pathways in oat genotypes with contrasting drought tolerance, we aim to determine whether this mechanism is endogenously employed by the plant. Such insights could inform breeding strategies that enhance drought resilience by leveraging intrinsic regulatory systems.

## 2 Materials and Methods

### 2.1 Plant Material, Growth Conditions and Sampling

Experiments were carried out using the oat cultivars Flega and Patones, supplied by the Andalusian Network of Agriculture Experimentation (RAEA). The physiology and the drought responses of these cultivars have been extensively described under controlled and field conditions (Canales, Montilla‐Bascón, Bekele, et al. 2021; Canales, Montilla‐Bascon, et al. 2019; Montilla‐Bascon et al. 2013; Sanchez‐Martin et al. 2015; Sanchez‐Martin, Rubiales, et al. 2012). The National Plant Genetic Resources Centre (INIA) provided an additional subset of five Spanish oat accessions, Gen16, Gen17, Gen100, Gen122, and Gen76, and two commercial cultivars, Alcudia and Anchuela, were provided by the RAEA. The responses to drought by this additional oat subset have previously been reported in Sanchez‐Martin, Mur, et al. (2012).

For this study, 3‐week‐old seedlings were used. Seedlings were grown in 0.75 L pots filled with peat: sand (2:1) in a growth chamber under 12/12 h dark/light periods with 250 μmol m^−2^ s^−1^ photon flux density supplied by white fluorescent tubes (Osram Licht AG), with 65% relative humidity and 20°C of temperature. Plants were watered regularly until they had two developed leaves and the third unrolled. At this time, water was withheld from those plants selected for drought treatment while control plants were watered regularly. At the end of the experiment (day 18), the drought treatments were at 20% sRWC (soil relative water content). No significant differences were observed in the soil water content between genotypes during the drought treatment (Figure S1), indicating exposure to similar water stress. Daily monitoring of sRWC following water withdrawal noted different levels at different times, which were reflected in sampling times *viz* mild water deficit (7 days after withholding water (daww), 55%–60% sRWC), moderate water deficit (9 daww, 40%–45% sRWC), high water deficit (11 daww, 30%–35% sRWC), and severe water deficit (14 daww; 20%–25% sRWC). The second leaf of each plant from the different genotypes and treatments was sampled.

### 2.2 In Vivo Gas Measurements in Intact Plants

NO and ethylene production were measured in intact oat plants (Flega and Patones cultivars) during the drought period using highly sensitive laser‐based detection systems.

NO production was monitored using a quantum cascade laser (QCL)‐based spectrometer equipped with an astigmatic multipass absorption cell for wavelength modulation spectroscopy (Sensor Sense B.V. and Trace Gas Facility, Nijmegen, The Netherlands) as described previously (Cristescu et al. 2013; Montilla‐Bascon et al. 2018; Montilla‐Bascon et al. 2017). The QCL emits in the mid‐infrared region around 1900 cm^−1^ and passes through the absorption cell, where NO is detected by measuring the attenuation of laser intensity due to NO absorption. To prevent the growth of microorganisms that could modify the NO balance, we used autoclaved soil to grow the plants. Soil microbial activity is a well‐documented and quantitatively significant source of NO emissions, particularly due to nitrifying and denitrifying microorganisms (Pilegaard 2013), whereas microorganisms on seed surfaces or in ambient air are not known to contribute significantly to NO production under the conditions used in our study. Therefore, autoclaving the soil was considered sufficient to minimize microbial interference and ensure that the NO measured originated primarily from plant physiological processes. The NO detector was calibrated using 100 ppbv NO in nitrogen (National Measurement Institute, Delft, the Netherlands). Data analyses were based on the LabVIEW program (National Instruments). In each experiment, four glass cuvettes (150 mL volume) containing one oat seedling each were measured sequentially. Each measurement cycle included a well‐watered tolerant plant and a well‐watered susceptible plant, and correspondingly, tolerant and susceptible plants where water had been withdrawn. Each experiment was repeated four times, with the order of plant genotype × treatment combination randomized to avoid measurement bias. NO accumulation was recorded over a 75‐min period in sealed cuvettes, after which the cuvettes were connected to the QCL system and flushed with hydrocarbon‐free air at a continuous flow rate of 1.66 L h^−1^.

Ethylene production was monitored in real time using a gas flow‐through in‐line system fitted with a laser‐based photoacoustic ethylene detector (ETD‐300, Sensor Sense, the Netherlands), which incorporates a QCL‐based spectrometer capable of detecting ethylene at concentrations as low as 300 parts per trillion by volume (pptv) within 5 s (Cristescu et al. 2013). Each seedling was placed in a 254 mL glass cuvette, and four cuvettes (representing the same genotype × treatment combinations as above) were monitored sequentially for 15 min (5 s per acquisition point) at a flow rate of 1.5 L h^−1^. Gas flow and cuvette switching were controlled by an automated valve system (VC‐6, Sensor Sense). KOH and CaCl_2_ scrubbers were used to remove CO_2_ and H_2_O, respectively, from the air stream before entering the detector. Background ethylene levels from autoclaved soil were minimal and subtracted from the final measurements.

### 2.3 Visual Assessment of Drought Symptoms

All plants were visually evaluated daily according to the following scale: 0 = vigorous plant, with no leaves showing drought symptoms; (1) one or two leaves (older leaves) show slight drought symptoms in the tips (less turgor) but most leaves remain erect; (2) several leaves show a slight decrease in turgor; however, most of the leaves show no drought symptoms; (3) most leaves show bending of the tip, although the rest of the leaf remains turgid, incipient yellowing of the older leaf; (4) all leaves show drought symptoms, including incipient wilting and/or yellowing of the older leaves; (5) all leaves start to appear rolled and/or shrunken (Sanchez‐Martin et al., 2015). The area under the drought progress curve (AUDPC) was calculated with the drought severity values daily assessed using the formula:

$$\text{AUDPC}=\sum \mathrm{ki}=1\;½\;\left[\left(\mathrm{Si}+\mathrm{Si}+1\right)\left(\mathrm{ti}+1-\mathrm{ti}\right)\right]$$

where Si is the drought severity at assessment date, ti is the number of days after the first observation on assessment date i, and k is the number of successive observations carried out during the days after withholding water. Assessments were performed on 10 independent plants per genotype and treatment.

### 2.4 Polyamine Quantification

Polyamine content was determined in control and droughted plants. Polyamine standards, putrescine (Put), spermidine (Spd), and spermine (Spm) were obtained as their hydrochlorides (Sigma). The second leaf of each plant was sampled, fixed in liquid nitrogen, and stored at −80°C until use. Plant tissue was homogenized in perchloric acid (0.1 w/v) (Flores and Galston 1982). Afterwards, standards and plant extracts were benzoylated (Redmond and Tseng 1979). High performance liquid chromatography (HPLC) analysis of benzoyl‐PAs was performed according to the method of Slocum et al. (1989), using an Agilent 1200 Series HPLC, including 1,7‐diaminoheptane (HTD) as the internal standard (Sigma). Five independent samples per genotype and treatment were analyzed.

To further support the effect of NO on polyamine production, polyamine content was assessed in non‐treated and 400 μM sodium nitroprusside (SNP; Fisher Scientific) treated leaves. Second leaves of Flega and Patones plants at a similar developmental stage to those at 14 daww were excised and incubated under a 12/12‐h dark/light cycle in the SNP or control solution for 72 h and then fixed and frozen in liquid nitrogen until polyamine assessment.

### 2.5 Gene Expression Analysis

#### 2.5.1 Primer Design

All primer sequences used for RT‐PCR are listed in Table 1. Primers for the arginine decarboxylase (*ADC*) gene were designed using the Universal Probe Library Assay Design Center (Roche Applied Science) based on mRNA sequences deposited in GenBank. To obtain *ACO5* sequence, an alignment of 11 *ACC* oxidase mRNA sequences on 4 oat‐related species (http://www.ncbi.nlm.nih.gov/BLAST/ [accessed on 22/12/2023]), (Table 2) was carried out to obtain a consensus sequence using the ClustalW method alignment in Bioedit software (ver. 7.0.5.3). The derived PCR product was purified with Qiagen QIAquick PCR Purification Kit and sequenced by STAB VIDA (https://www.stabvida.com.es). The sequence was deposited into the National Center for Biotechnology Information (NCBI) GenBank database, accession number PQ423727.1. This sequence was used to design the *ACO5* oat primers (Table 1) using the Universal Probe Library Assay Design Center. Primers originally developed for the barley phytoglobin *HvPgb1* (formerly *HvHb*1) (Hebelstrup et al. 2014) successfully amplified oat *Pgb* homologue (*AsPgb*) and were therefore used for our expression study. The specificity of all primers was checked by alignments with the original GenBank sequences using the standard nucleotide–nucleotide BLAST (blastn; provided online by NCBI http://blast.ncbi.nlm.nih.gov/Blast.cgi).

**TABLE 1:** Primer sequences.

Primer	Sequence	Length (bp)
ACO5Oat_Fw	GGACGACATGGACTGGGAG	19
ACO5Oat_Rv	GCCTCGAAGGCCGGGTTGT	19
ADCOat_Fw	TACGGCGATGTGTACCATGT	20
ADCOat_Rv	GTCCTTGTTCACGGCAAAGT	20
HvHB1_Fw	TCGTCTTCAGCGAGGAGAAG	20
HvHB1_Rv	GATCTCGAAGATCTTGAGGAAG	22

Abbreviations: *ACO*5, 1‐aminocyclopropane‐1‐carboxylic acid Oxidase; *ADC*, arginine decarboxylase; barley phytoglobin *HvPgb1* (formerly non‐symbiotic hemoglobin *HvHB1* gene) primer sequences and RT‐PCR conditions.

**TABLE 2:** ACC oxidase mRNA sequences. Identification of ACC oxidase mRNA sequences aligned to obtain ACO5 primers.

Accesion number	Query ID	Specie
gi|397740901	JX046062.1	*Hordeum vulgare*
gi|326526920	AK369648.1	*Hordeum vulgare*
gi|241990219	AK330561.1	*Triticum aestivum*
gi|397740903	JX046063.1	*Hordeum vulgare*
gi|326510640	AK356319.1	*Hordeum vulgare*
gi|326509860	AK355928.1	*Hordeum vulgare*
gi|151421294	AK248278.1	*Hordeum vulgare*
gi|242385394	FP093445.1	*Phyllostachys edulis*
gi|242386752	FP099157.1	*Phyllostachys edulis*
gi|242386837	FP099242.1	*Phyllostachys edulis*
gi|115462114	NM_001061192.1	*Oryza sativa*

#### 2.5.2 RNA Extraction and cDNA Amplification

Total RNA was extracted from 100 mg of ground leaf tissue using previously reported protocols (Chomczynski and Sacchi 1987; Raeder and Broda 1985) and purified using the RNeasy Minelute Cleanup Kit (QIAGEN). The absence of any residual genomic DNA in samples was verified by a failure to PCR amplify from total RNA. RNA samples containing DNA were DNase‐treated until there was no PCR amplification from the RNA samples. The concentration and integrity of RNA were verified based on optical density at 260 nm (OD260)/OD280 absorption ratio in a NanoDrop ND‐1000 spectrophotometer (Thermo Scientific).

SuperScript III First‐Strand (Invitrogen) and DNA Polymerase I (BioLabs) were used to synthesize the first and second strands of complementary DNA (cDNA), respectively. The cDNA was purified using a QUIquick PCR Purification Kit (Qiagen). The quality and quantity of cDNA were determined by running PCR amplified samples on agarose gels and by spectrophotometric analysis in a NanoDrop ND‐1000 spectrophotometer (Thermo Scientific).

#### 2.5.3 Gene Expression Analysis by Real‐Time RT‐PCR

Four genes were tested for their value as reference genes, beta‐tubulin (*TUBB*), alpha‐tubulin (*TUBA*), and 18S ribosomal RNA (18S rRNA) according to Jarosova and Kundu (2010) and also glyceraldehyde 3‐phosphate dehydrogenase (*GADPH*). Of these, GADPH was selected as an internal control since it showed similar levels of expression in all of our oat samples. Real‐time qRT‐PCR was performed for *ADC, ACO5*, and *Pgb* genes on at least 3 independent biological plus three technical replicated cDNA templates in the StepOne Real‐Time PCR System (Applied Biosystems) using FastStart Universal SYBR Green Master (Rox) (Roche) according to the manufacturer's recommendations. The reaction mixture contained 10 μL of SYBR Green master mix, 6 μL of primers (0.3 μM final concentration), and 4 μL of cDNA or standard solution as template. The amplification conditions were 95°C for 10 min, followed by 40 cycles of amplification at 95°C for 15 s and 60°C for 1 min. Following amplification, a melting curve program of 95°C for 15 s, 60°C for 1 min, and 60°C–95°C at a heating rate of 0.3°C min^−1^ was performed. To assess the copy number of the target genes, we examined the three available oat reference genomes *(OTpepsico_v2* (Pepsico 2021), *Sang* (Kamal et al. 2022), and *Sanfensan* (Peng et al. 2022)). *AsPgb1* was found to be present as a single‐copy gene on chromosome 1A. *ADC* homologues were identified on chromosomes 4C, 5D, and 6A, with more than 88% sequence identity. Notably, seven nearly identical *ADC* homologues are arranged in tandem on chromosome 5D, all targeted by our primers. *ACO5* homologues were detected in tandem duplications on chromosomes 1D, 3D, and 7A, sharing over 98% identity and also amplified by our *ACO5* primers. To confirm reaction specificity, we monitored the melting curves of the qPCR amplicons, which consistently revealed a single peak for each gene, indicating the amplification of unique products. The fold changes were derived according to the 2^−∆∆Ct^ method (Livak and Schmittgen 2001).

### 2.6 Leaf Chlorophyll Content

A SPAD‐502 chlorophyll meter (Minolta Co. LTD.) was used to estimate leaf chlorophyll content in plants (Sanchez‐Martin et al., 2012a). Measurements were taken 6 h after the onset of the light period with the adaxial side placed toward the emitting window of the instrument. The derived measurement represented the mean of three meter readings for each leaf.

### 2.7 Statistical Analysis

Experiments followed a completely randomized design. For ease of understanding, means of raw percentage data are presented in the figures. However, to normalize data and stabilize variances, percentage data were arcsine square roots (transformed value = 180/п × arcsine [√(%/100)]). Data were subjected to ANOVA analysis of variance using SPSS software, and residual plots were inspected to confirm that the data conformed to normality. In addition, the significance of differences between means was determined by contrast analysis (Scheffe's).

## 3 Results

Drought treatment was applied to the two oat cultivars with contrasting responses to drought, Flega and Patones, respectively, susceptible and tolerant. Where water was withdrawn, sRWC was gradually reduced over the timeframe of the experiment to reach 20%. At this stage, the plants showed no sign of wilting. A visual assessment of symptoms showed that seedlings exhibited the expected responses to drought (Figure 2A,B) (Sanchez‐Martin, Mur, et al. 2012; Sanchez‐Martin et al. 2014, 2015, 2018; Canales, Montilla‐Bascon, et al. 2019; Canales, Nagel, et al. 2019; Canales, Montilla‐Bascón, Gallego‐Sánchez, et al. 2021; Canales, Rispail, Garcia‐Tejera, et al. 2021; Girija et al. 2025).

### 3.1 In Vivo NO Measurements Show Increased Endogenous NO Production in Drought‐Susceptible Flega Plants

**FIGURE 1:**
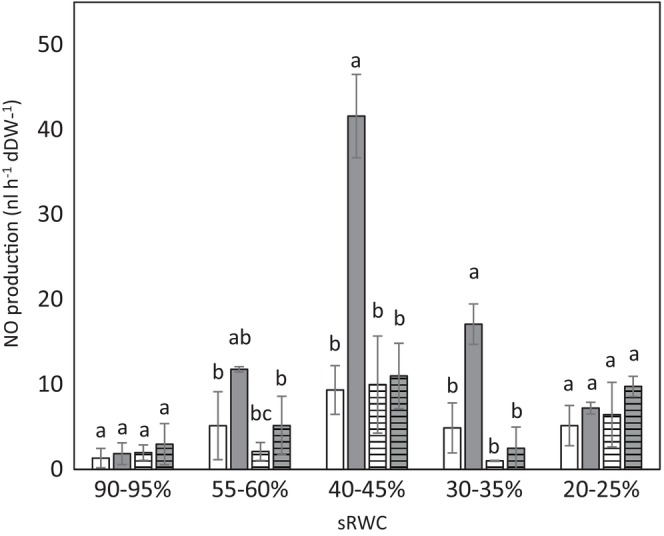
In vivo nitric oxide (NO) production in Flega and Patones oat plants under drought. NO was quantified in susceptible Flega (solid bars) and tolerant Patones (hatched bars) well‐watered plants (white) and during a time course of water stress (grey) at different soil relative water content. Data are the mean of four replicates ± standard error. For each sRWC, different letters indicate significant differences at *p* < 0.05 between genotypes and treatments.

QCL‐based spectrometry was used to assess in vivo NO production in oat plants under well‐watered and droughted conditions (Figure 1). No significant differences were observed in NO levels between well‐watered control plants of either cultivar. However, under drought conditions, clear differences emerged both over the progression of stress and between genotypes. Drought‐susceptible cv. Flega showed significantly increased endogenous NO production under 50%–60% sRWC compared to its well‐watered controls. The highest NO levels were observed at 40%–45% sRWC in Flega, showing a nearly 4‐fold increase compared to Patones at the same water status. Whilst NO levels in Flega declined slightly at 30%–35% sRWC, they remained significantly elevated relative to controls. At 20%–25% sRWC, NO production in Flega was no longer significantly different from well‐watered controls. In contrast, drought‐tolerant Patones maintained NO levels comparable to its controls throughout the drought time course, showing no significant increase in NO under water stress.

### 3.2 Patones Exhibits Increased Phytoglobin ( *AsPgb* ) Gene Expression With Progressive Water Stress

**FIGURE 2:**
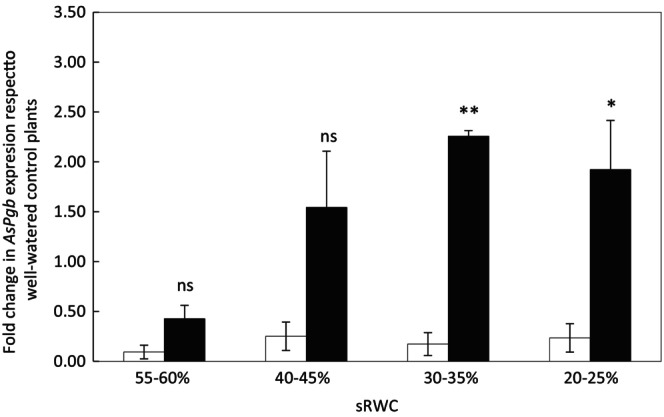
Expression of phytoglobin (*AsPgb*) gene in oat plants under drought conditions. Expression of *AsPgb* was measured during a drought time course in susceptible Flega (white bars) and resistant Patones (black bars) plants. Data are mean of at least three independent biological plus three technical replications ± standard error. *, ** and ns indicate significant differences at *p* < 0.05 and 0.01 and non‐significant differences, respectively between Flega and Patones.

Under mild water stress (55%–60% RWC), there were no significant differences in *AsPgb* expression between Flega and Patones plants, with both genotypes showing slightly downregulated levels compared to their well‐watered controls (Figure 2). However, while *AsPgb* expression in Flega was downregulated compared to its well‐watered control for the whole drought time‐course (*p* = 0.012), Patones exhibited a significant increase in expression from 30% to 35% sRWC compared to well‐watered plants. Subsequently, expression in Patones plants remained significantly higher than in Flega, with *AsPgb* expression in Patones being over 13‐fold higher than in Flega. Overall, there were significant effects of genotype (*p <* 0.001) and treatment (*p =* 0.037), highlighting a differential transcriptional response to drought between the two cultivars, with tolerant Patones showing overexpressed levels of the phytoglobin gene.

The measured levels of NO are expected to reflect not solely the activity of *AsPgb*, but a dynamic balance between NO synthesis and scavenging. In line with this, a correlation analysis between NO and *AsPgb* expression revealed a negative trend across the full time course (*r* = −0.32); however, this became stronger at intermediate stress stages (*r* = −0.40) and became highly significant at 40%–45% sRWC (*r* = −0.98, *p* = 0.0032). This supported a context‐dependent role of phytoglobins in NO modulation.

### 3.3 Water Stress‐Mediated NO Increases Correlate With Polyamine Pathway Changes With Increases in Discrete Polyamines

**FIGURE 3:**
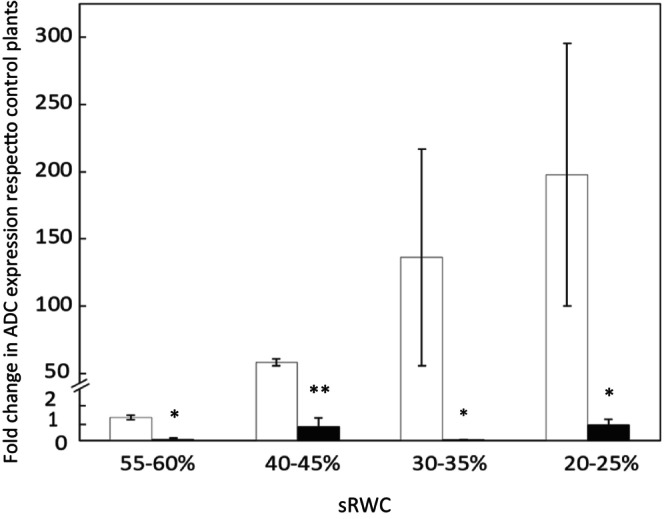
Expression of arginine decarboxylase (*ADC*) involved in the polyamine pathway in oat under drought conditions. Expression of *ADC* was measured during a drought time course in Flega (white bars) and Patones (black bars) plants. Data are mean of at least three independent biological plus three technical replications ± standard error. *, ** indicate significant differences at *p* < 0.05 and 0.01, respectively between Flega and Patones.

To explore the potential link between NO and polyamines in oats suffering from water deficit, *ADC* expression was measured in Patones and Flega (Figure 3). While no significant changes were observed in Patones between droughted and well‐watered plants, Flega showed consistently higher *ADC* expression throughout the drought time course, both in comparison to its control (*p =* 0.04) and to Patones (*p =* 0.004). The differences between cultivars ranged from over 16‐fold to more than 1000‐fold depending on the stress severity, suggesting more pronounced polyamine biosynthesis in the susceptible genotype.

**FIGURE 4:**
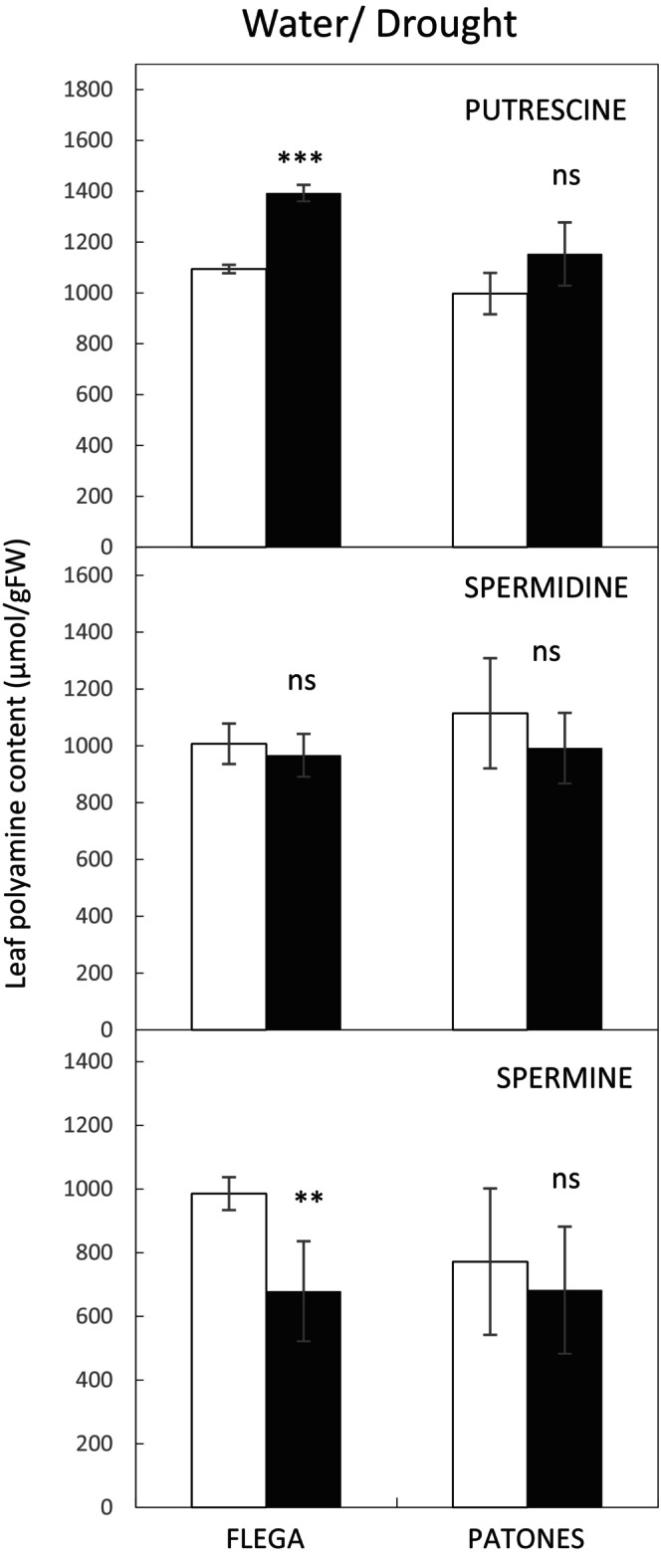
Polyamine content in Flega and Patones under drought. Putrescine, spermidine and spermine were measured at 20%–25% soil relative water content in Flega and Patones plants. White bars = control, well‐watered plants; Black bars = plants exposed to water stress. Data are mean of five replicates ± standard error. **, *** and ^ns^indicate significant differences between droughted and well‐watered plants at *p* < 0.01, 0.001 and non‐significant differences, respectively.

Next, the levels of Put, Spd, and Spm were determined at 20% sRWC (Figure 4). Flega exhibited highly significant increases in Put compared to watered controls (*p <* 0.001; Figure 4). However, the levels of a “downstream” polyamine, Spm, were significantly reduced. The levels of Put, Spd, and Spm were not significantly affected by drought in Patones (Figure 4).

**FIGURE 5:**
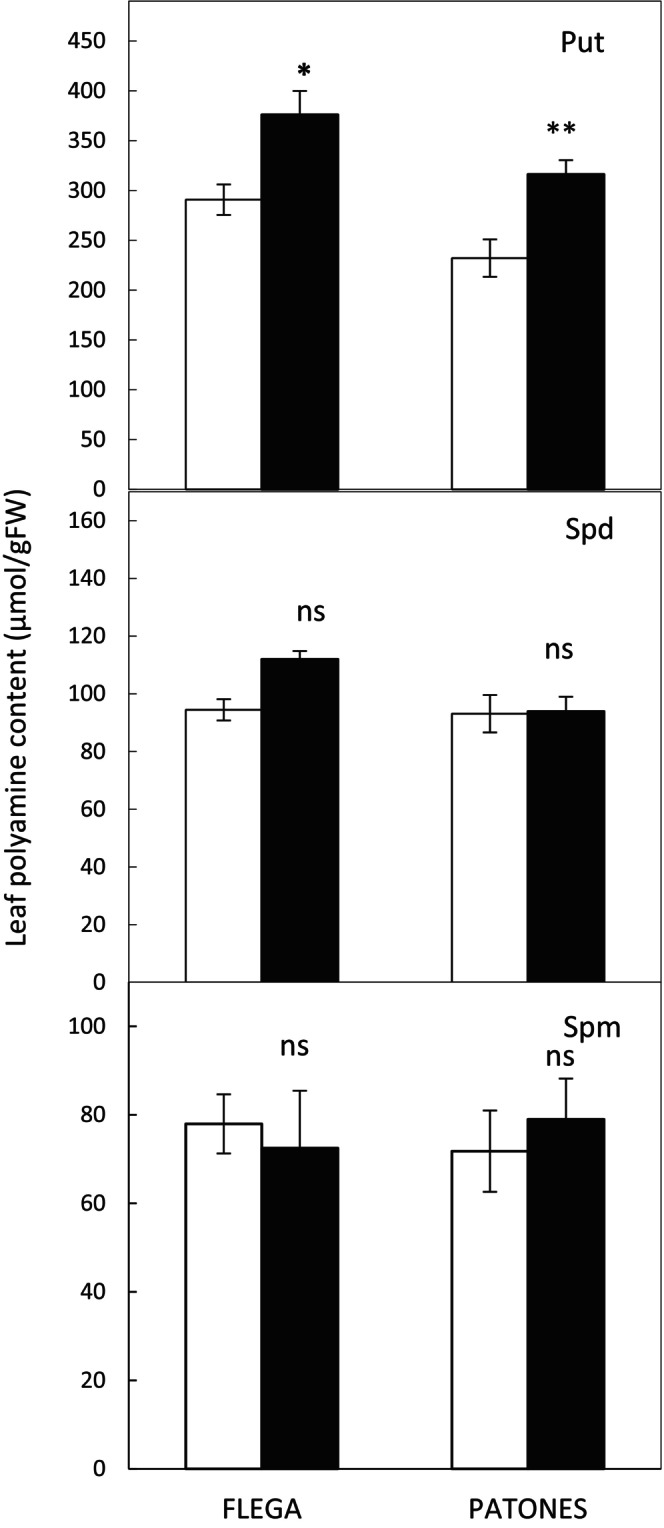
Effect of the NO donor sodium nitropusside (SNP) in polyamine content in Flega and Patones under drought. Putrescine (Put), spermidine (Spd) and spermine (Spm) were measured at 20%–25% soil relative water content in Flega and Patones plants. White bars = control, well‐watered plants; Black bars = plants exposed to water stress. Data are mean of five replicates ± standard error. *, ** and ^ns^indicate significant differences between droughted and well‐watered plants at *p* < 0.05, 0.01 and non‐significant differences, respectively.

To support the hypothesized link between NO changes and polyamine biosynthesis, oat leaves were treated with the NO donor SNP. Following SNP treatment, there were significant increases in the levels of Put in both Flega and Patones plants (Figure 5). However, there were no significant changes in Spd or Spm.

### 3.4 Increased Levels of Ethylene and Drought‐Related Senescence Symptoms Are Associated With Naturally Increased NO Levels in Susceptible Cultivar Flega

**FIGURE 6:**
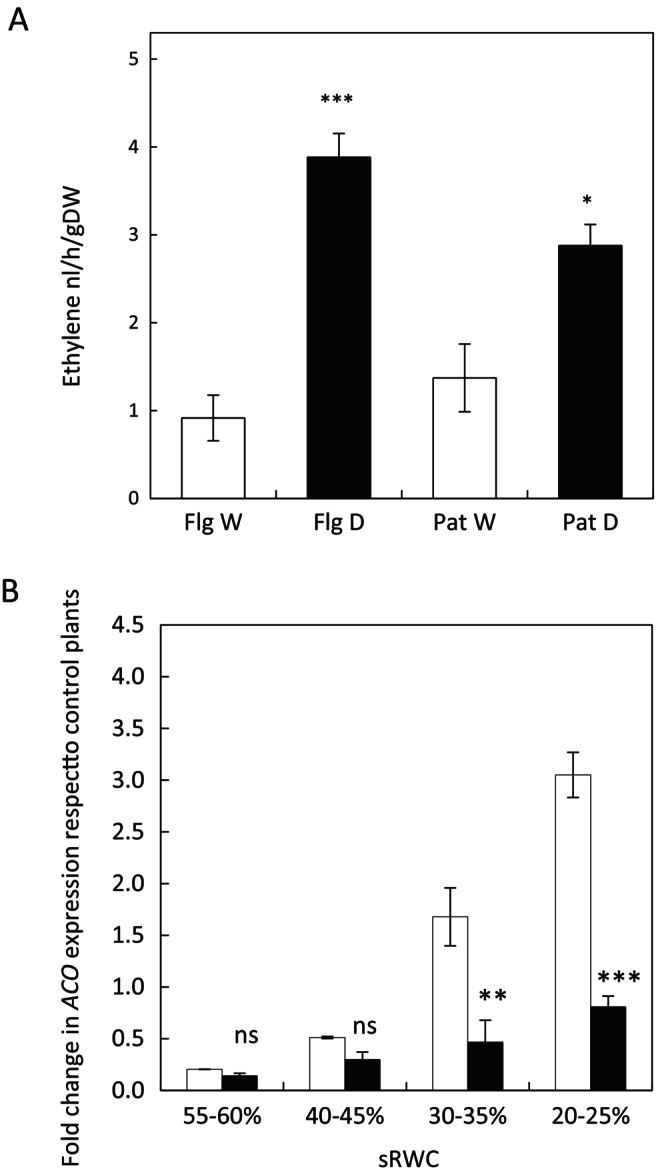
In vivo ethylene production and *ACO* expression in Flega and Patones plants. (A) In vivo ethylene generation in Flega (Flg) and Patones (Pat) oat plants were quantified in whole intact oat plants, well watered (white bars) or at 20%–25% sRWC (black bars). Data are mean of four replicates ± standard error. (B) Expression of 1‐aminocyclopropane‐1‐carboxylic acid oxidase (*ACO5*) gene was measured during a drought time course in Flega (white bars) and Patones (black bars) plants. Data are mean of at least three independent biological plus three technical replications ± standard error. **, *** and ns indicate significant differences with respect well‐watered plants at *p* < 0.01 and 0.001, and non‐significant differences, respectively.

To investigate whether ethylene‐related changes are an inherent feature of oats under drought, in vivo ethylene emissions were measured in Flega and Patones plants under well‐watered and drought conditions (Figure 6). Ethylene production was significantly influenced by genotype (*p* < 0.05), treatment (*p* < 0.001), and their interaction (*p <* 0.05). Under drought conditions, ethylene generation increased significantly in both Flega and Patones plants under drought stress, but levels were significantly higher in Flega, which produced nearly 1.5 times more ethylene than Patones under drought (Figure 6A). Importantly, ethylene production under severe drought stress showed a significant positive correlation with in vivo NO levels at 30%–35% sRWC (*r* = 0.68, *p =* 0.02).

These patterns of ethylene production were supported by the expression of *ACO5* (Figure 6B). Flega showed progressively higher *ACO5* expression than Patones as drought intensified, with fold differences ranging from 1.4‐fold at 55%–60% sRWC to nearly 3.8‐fold at 20%–25% sRWC. Notably, Patones maintained downregulated *ACO5* expression compared to its well‐watered control throughout the drought time course (*p =* 0.009), suggesting genotype‐specific regulation of ethylene biosynthesis under stress.

**FIGURE 7:**
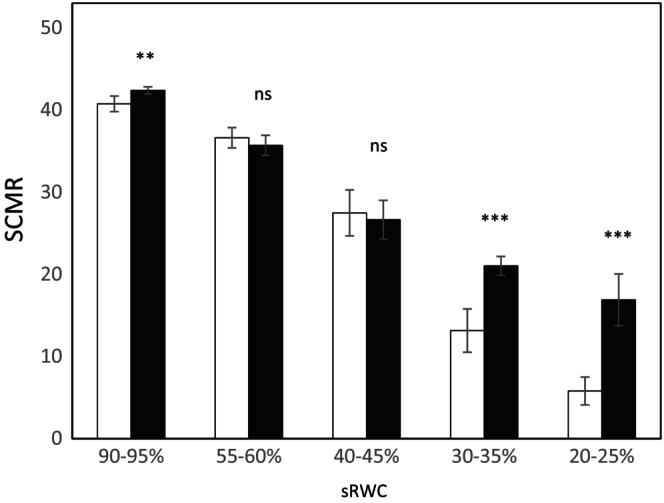
Spad chlorophyll meter readings (SCMR) of oat Flega and Patones cultivars. SCMR were assessed at 20%–25% sRWC in Flega (triangle) and Patones (circles) plants. Data are based on five replicates per genotype and treatment and three SCMR per leaf. ** and *** indicate significant differences at *p* < 0.01, 0.001, and ^ns^indicate non‐significant differences with respect to control plants.

Ethylene has been associated with stress‐induced leaf senescence. To determine whether the ethylene increase observed in droughted plants was linked to early senescence, leaf chlorophyll content was measured using a SPAD meter (Figure 7). Flega plants had significantly lower chlorophyll content than tolerant Patones plants under drought, with values approximately 1.6‐fold lower at 30%–35% sRWC and nearly 3‐fold lower at 20%–25%. These results are consistent with the visual symptom scale, which indicated more severe drought symptoms in Flega plants under water stress (Figure S1).

### 3.5 Considering the Potential Role of Phytoglobin Expression in Conferring Drought Tolerance in Other Oat Genotypes

Taken together, our data suggested that increased *AsPgb* expression was important in conferring drought tolerance in Patones. To explore whether this pattern was consistent across other oat genotypes previously characterised for drought response according to 28 physiological variables (Sanchez‐Martin et al. 2015; Sanchez‐Martin, Mur, et al. 2012) and under field conditions (Canales, Montilla‐Bascón, Gallego‐Sánchez, et al. 2021). These studies indicated that cultivar Alcudia was highly susceptible to drought (similar to Flega), cultivars Mirabel, Anchuela, and accessions Gen16, Gen76, and Gen122 were moderately susceptible, and accessions Gen17 and Gen100 were moderately tolerant. No accessions were as tolerant to drought as Patones.

Each accession was subjected to the same progressive drought treatment or watered conditions as had been previously used with Flega and Patones. Endpoint *AsPgb* expression analyses were undertaken when sRWC was 20%–25%. *AsPgb* expression in these oat genotypes showed significant differences between genotypes, treatments, and the interaction between these factors, indicating that *AsPgb* regulation under drought is genotype‐dependent. The highly susceptible cultivar Alcudia showed a marked and significant downregulation of the *AsPgb* gene under drought, with expression reduced to less than half of control levels. A similar trend was observed in moderately susceptible genotypes such as Anchuela, Mirabel, Gen16, Gen76, and Gen 122, all showing reductions ranging from 40% to 80% compared to their respective controls.

In contrast, the moderately tolerant accessions Gen17 and Gen100 both exhibited a significant upregulation of *AsPgb* expression under drought, with increases of around 2‐fold compared to their respective controls, resembling the pattern observed in Patones (Figure 2). Overall, *AsPgb* expression under drought showed a significant negative correlation with drought symptoms (*r* = −0.512, *p* < 0.001) between *AsPgb* expression and drought plant symptoms as assessed using the visual score method.

## 4 Discussion

The role of NO in plant drought responses has elicited significant attention in recent years, with numerous studies attempting to unravel its mechanism of action and interactions with other signalling molecules (reviewed in Lau et al. 2021). Despite this, the exact functions of NO in drought responses remain unclear, with conflicting findings across various studies. Many studies have based their findings on the use of NO donors such as SNP or GSNO. For instance, Lau et al. (2021) list 33 out of 35 NO studies on drought stress that used exogenously applied SNP or GSNO at different concentrations. None of them monitored inherent NO changes under drought stress, and many of them showed divergent results, for example, Batista et al. (2018), Casaretto et al. (2021), Farouk and AL‐Huqail (2020), Pissolato et al. (2020), Rigui et al. (2019), Sahay et al. (2019), so the use of NO donors alone may not accurately reflect physiological events. Our previous research has extensively characterised in vivo patterns of NO production and used the over‐expression of the *Pgb* gene in barley to reduce its endogenous levels to increase drought tolerance (Montilla‐Bascon et al. 2017). This study suggested a complex interaction between NO, ethylene, and polyamine metabolism conferring drought susceptibility or tolerance (Montilla‐Bascon et al. 2017). However, manipulating specific genes or metabolites may not fully capture or reveal the natural plant strategy to respond to stress.

Here, we sought to assess how far the mechanisms defined in barley transgenic plants could also be relevant to oats. In line with this, the susceptible oat cultivar Flega exhibited increased NO production during a gradual water deficit time course, with most production observed under moderate stress. Conversely, the tolerant cultivar Patones shows no changes in NO compared to well‐watered controls. This was consistent with a negative role for NO in drought tolerance, as also seen with barley (Montilla‐Bascon et al. 2017). Further, the increase of NO in the susceptible cultivar was also associated with increases in *ADC* and Put as well as *ACO5*, ethylene, and early senescence, features also described in barley (Montilla‐Bascon et al. 2017). These findings differ from others, which suggested that increases in NO mitigate drought symptoms (Adamipour et al. 2020; Ahammed et al. 2021; Akin and Kaya 2024; Imran et al. 2021; Lian et al. 2023; Rezayian et al. 2023; Ruan et al. 2024), but the difference could be related to the relative extent of stress, timing, the plant species involved, or discrete drought response strategies.

One of the most widely studied actions of NO in drought responses is the induction of stomatal closure, mediated by increases in stomatal cytoplasmic pH and H_2_O_2_ levels (Bright et al. 2006; Neill et al. 2002). However, it would be difficult to exploit a drought tolerance mechanism based on stomatal closure in plant breeding as CO_2_ uptake would be compromised (Blum 2009, 2015; Galmes et al. 2013, 2007). This is supported by studies on wheat, rice, or cotton genotypes where genotypes with higher yields under drought stress typically exhibit greater stomatal conductance (Araus et al. 2002; Blum 2009; Izanloo et al. 2008). Flega experiences a rapid and significant increase in abscisic acid levels under drought, resulting in a rapid reduction of stomatal conductance, but leaf water potential decreases due to a decline in root hydraulic conductivity, leading to oxidative damage (Canales, Rispail, Garcia‐Tejera, et al. 2021). In contrast, Patones is characterised by a gradual and moderate rise in abscisic acid, allowing for prolonged transpiration, linked to an increase in root hydraulic conductivity, achieved through increased total root length and the elongation of finer roots that enhances root conductivity (Canales, Rispail, Garcia‐Tejera, et al. 2021). These adjustments avoid oxidative stress and enable the resistant cultivar to uphold higher water potential, mitigating the impacts of drought and promoting growth under water deficit conditions in the field and under controlled conditions (Canales, Nagel, et al. 2019; Sánchez‐Martín et al. 2018, 2015; Sanchez‐Martin, Mur, et al. 2012). Given these observations, it is important that our proposed mechanism of drought tolerance is based on the differential expression of *Pgb*. Although transcript levels do not always mirror protein abundance due to post‐transcriptional and translational regulation, the expression patterns of *AsPgb* observed in this study align with physiological changes in NO levels, particularly under critical stress conditions. The strong inverse relationship between *AsPgb* expression and NO under critical stress levels of 40%–45% sRWC suggests that phytoglobins are actively involved in modulating NO levels during certain drought conditions.

Mechanistically, phytoglobin could be countering NO levels induced increases in Put production via *ADC* overexpression. Previous studies have shown that the ADC protein undergoes post‐translational cleavage under osmotic stress, prompting the formation of Put (Malmberg et al. 1992; Trull and Malmberg 1994). Elevated levels of Put in leaves lead to chlorophyll loss and accelerated senescence (Capell et al. 1998), characteristics that we observed in Flega during water stress. It will be interesting to assess how NO‐mediated modifications of ADC, such as *S‐nitrosylation*, could be influencing the change in flux to Put synthesis. Phytoglobin could also be countering NO‐induced senescence through suppression of ethylene production. Our results showed a significant correlation between the in vivo NO increases and in vivo increases in ethylene production via overexpression of *ACO5*, with significantly higher levels in the susceptible genotype. This matched reports of an increase in ethylene levels and the expression of *ACS* and *ACO* genes during water stress to enhance drought symptoms, that is, Arraes et al. (2015).

These findings are consistent with the NO–PGB1 link described in maize under PEG‐simulated water deficit to promote tolerance (Hammond et al. 2020) and more specifically to the NO‐PGB1‐ethylene regulatory axis proposed by Hartman et al. (2019) and further discussed by Ugalde (2022), in the context of hypoxia tolerance. Hartman et al. (2019) did observe variation in the NO‐PGB1‐ethylene effects between genotypes. Similarly, our data suggest a genotypic variation in the NO‐PGB1‐ethylene effects with drought. Patones showed a correlation between ethylene and the upregulation of *AsPgb*, supporting a link between ethylene, NO scavenging, and stress adaptation. In contrast, Flega fails to show *AsPgb* induction under stress, which may contribute to its susceptibility.

**FIGURE 8:**
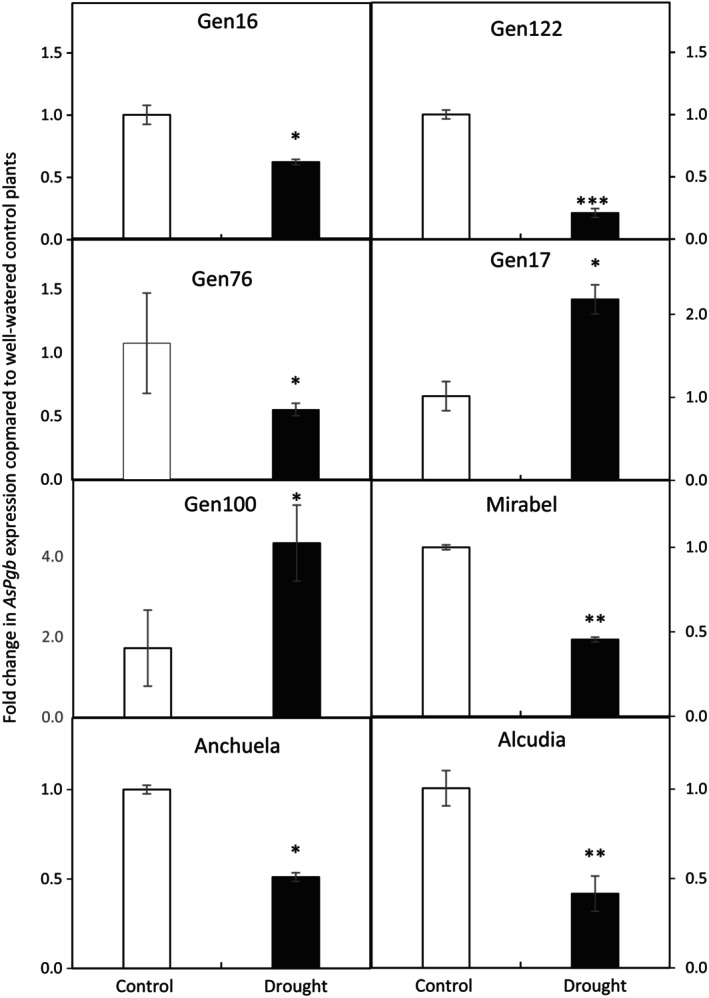
Expression of phytoglobin *(AsPgb)* gene in an additional set of oat genotypes under drought conditions. Expression of *AsPgb* was measured at 20%–25% sRWC in an additional set of eight oat accessions under well‐watered conditions (white bars) or water stress (black bars). Data are mean of at least three independent biological plus three technical replications ± standard error. *, **; *** and ^ns^indicate significant differences at *p* < 0.05, 0.01, and 0.001 and non‐significant differences, respectively.

Taking all of our observations together, the differential expression of the *AsPgb* gene between susceptible and resistant cultivars reveals a promising target for genetic improvement. By selecting for genotypes that show upregulated *Pgb* under drought conditions (such as shown in Figure 8), it may be possible to develop varieties with higher drought resistance. Moreover, understanding the interplay between NO scavenging, ethylene reduction, and polyamine metabolism offers valuable insights into plant stress physiology to identify additional targets for improving drought stress tolerance and enhancing crop performance in the face of increasing environmental challenges.

## Author Contributions

G.M.‐B. made most of the experimental work, data analysis, and writing of the manuscript. S.M.C. and L.A.J.M. contributed to in vivo NO and ethylene analysis, interpretation of data, and writing the manuscript. E.P. steered the research, designed experiments, contributed to the interpretation of results, and writing of the manuscript.

## Supporting information


**Data S1:** Supporting Information.

## Data Availability

The *ACO5* sequence generated in this study has been deposited into the National Center for Biotechnology Information (NCBI) GenBank database, accession number PQ423727.1. The data that support the findings of this study are available from the corresponding author upon reasonable request.

## References

[ppl70597-bib-0001] Adamipour, N. , M. Khosh‐Khui , H. Salehi , H. Razi , A. Karami , and A. Moghadam . 2020. “Role of Genes and Metabolites Involved in Polyamines Synthesis Pathways and Nitric Oxide Synthase in Stomatal Closure on *Rosa damascena* Mill. Under Drought Stress.” Plant Physiology and Biochemistry 148: 53–61. 31927272 10.1016/j.plaphy.2019.12.033

[ppl70597-bib-0002] Ahammed, G. J. , X. Li , Q. Mao , H. J. Wan , G. Z. Zhou , and Y. Cheng . 2021. “The SlWRKY81 Transcription Factor Inhibits Stomatal Closure by Attenuating Nitric Oxide Accumulation in the Guard Cells of Tomato Under Drought.” Physiologia Plantarum 172: 885–895. 33063343 10.1111/ppl.13243

[ppl70597-bib-0003] Akin, S. , and C. Kaya . 2024. “Asparagine and Nitric Oxide Jointly Enhance Antioxidant Capacity and Nitrogen Metabolism to Improve Drought Resistance in Cotton: Evidence From Long‐Term Field Trials.” Food and Energy Security 13: e502.

[ppl70597-bib-0004] Araus, J. L. , G. A. Slafer , M. P. Reynolds , and C. Royo . 2002. “Plant Breeding and Drought in C_3_ Cereals: What Should We Breed for?” Annals of Botany 89: 925–940. 12102518 10.1093/aob/mcf049 PMC4233799

[ppl70597-bib-0005] Arraes, F. B. M. , M. A. Beneventi , M. E. L. DE Sa , et al. 2015. “Implications of Ethylene Biosynthesis and Signaling in Soybean Drought Stress Tolerance.” BMC Plant Biology 15: 213–233. 26335593 10.1186/s12870-015-0597-z PMC4557918

[ppl70597-bib-0006] Batista, P. F. , A. C. Costa , C. Müller , et al. 2018. “Nitric Oxide Mitigates the Effect of Water Deficit in *Crambe abyssinica* .” Plant Physiology and Biochemistry 129: 310–322. 29925047 10.1016/j.plaphy.2018.06.012

[ppl70597-bib-0007] Blum, A. 2009. “Effective Use of Water (EUW) and Not Water‐Use Efficiency (WUE) is the Target of Crop Yield Improvement Under Drought Stress.” Field Crops Research 112: 119–123.

[ppl70597-bib-0008] Blum, A. 2015. “Towards a Conceptual ABA Ideotype in Plant Breeding for Water Limited Environments.” Functional Plant Biology 42: 502–513. 32480696 10.1071/FP14334

[ppl70597-bib-0009] Bright, J. , R. Desikan , J. T. Hancock , I. S. Weir , and S. J. Neill . 2006. “ABA‐Induced NO Generation and Stomatal Closure in Arabidopsis Are Dependent on H_2_O_2_ Synthesis.” Plant Journal 45: 113–122. 10.1111/j.1365-313X.2005.02615.x 16367958

[ppl70597-bib-0011] Canales, F. J. , G. Montilla‐Bascón , L. M. Gallego‐Sánchez , F. Flores , N. Rispail , and E. Prats . 2021. “Deciphering Main Climate and Edaphic Components Driving Oat Adaptation to Mediterranean Environments.” Frontiers in Plant Science 12: 1–15. 10.3389/fpls.2021.780562 PMC8662754 34899808

[ppl70597-bib-0012] Canales, F. J. , G. Montilla‐Bascon , N. Rispail , and E. Prats . 2019. “Salicylic Acid Regulates Polyamine Biosynthesis During Drought Responses in Oat.” Plant Signaling & Behavior 14: e1651183. 31382811 10.1080/15592324.2019.1651183 PMC6768256

[ppl70597-bib-0010] Canales, F. J. , G. Montilla‐Bascón , W. A. Bekele , et al. 2021. “Population Genomics of Mediterranean Oat (*A. sativa*) Reveals High Genetic Diversity and Three Loci for Heading Date.” Theoretical and Applied Genetics 134: 2063–2077. 33770189 10.1007/s00122-021-03805-2 PMC8263550

[ppl70597-bib-0013] Canales, F. J. , K. A. Nagel , C. Müller , N. Rispail , and E. Prats . 2019. “Deciphering Root Architectural Traits Involved to Cope With Water Deficit in Oat.” Frontiers in Plant Science 10: 1558. 31850037 10.3389/fpls.2019.01558 PMC6892839

[ppl70597-bib-0014] Canales, F. J. , N. Rispail , O. Garcia‐Tejera , V. Arbona , A. Perez‐De‐Luque , and E. Prats . 2021. “Drought Resistance in Oat Involves ABA‐Mediated Modulation of Transpiration and Root Hydraulic Conductivity.” Environmental and Experimental Botany 182: 1–14.

[ppl70597-bib-0015] Capell, T. , C. Escobar , H. Liu , D. Burtin , O. Lepri , and P. Christou . 1998. “Over‐Expression of the Oat Arginine Decarboxylase cDNA in Transgenic Rice *Oryza sativa* Affects Normal Development Patterns *in Vitro* and Results in Putrescine Accumulation in Transgenic Plants.” Theoretical and Applied Genetics 97: 246–254.

[ppl70597-bib-0016] Casaretto, E. , S. Signorelli , J. P. Gallino , S. Vidal , and O. Borsani . 2021. “Endogenous • NO Accumulation in Soybean Is Associated With Initial Stomatal Response to Water Deficit.” Physiologia Plantarum 172: 564–576. 33159328 10.1111/ppl.13259

[ppl70597-bib-0017] Chomczynski, P. , and N. Sacchi . 1987. “Single‐Step Method of RNA Isolation by Acid Guanidinium Thiocyanate‐Phenol‐Chloroform Extraction.” Analytical Biochemistry 162: 156–159. 2440339 10.1006/abio.1987.9999

[ppl70597-bib-0018] Cristescu, S. M. , J. Mandon , D. Arslanov , J. DE Pessemier , C. Hermans , and F. J. M. Harren . 2013. “Current Methods for Detecting Ethylene in Plants.” Annals of Botany 111: 347–360. 23243188 10.1093/aob/mcs259 PMC3579434

[ppl70597-bib-0019] De Sousa, L. F. , P. E. de Menezes‐Silva , L. L. Lourenco , et al. 2020. “Improving Water Use Efficiency by Changing Hydraulic and Stomatal Characteristics in Soybean Exposed to Drought: the Involvement of Nitric Oxide.” Physiologia Plantarum 168: 576–589. 31102278 10.1111/ppl.12983

[ppl70597-bib-0020] Ehlers, W. 1989. “Transpiration Efficiency of Oat.” Agronomy Journal 81: 810–817.

[ppl70597-bib-0021] FAO , and T. F. A. A. O . 2023. “*FAOSTAT* [Online].” https://www.fao.org/faostat/en/#data/QCL.

[ppl70597-bib-0022] Farouk, S. , and A. A. AL‐Huqail . 2020. “Sodium Nitroprusside Application Regulates Antioxidant Capacity, Improves Phytopharmaceutical Production and Essential Oil Yield of Marjoram Herb Under Drought.” Industrial Crops and Products 158: 113034.

[ppl70597-bib-0023] Flores, H. E. , and A. W. Galston . 1982. “Analysis of Polyamines in Higher‐Plants by High‐Performance Liquid‐Chromatography.” Plant Physiology 69: 701–706. 16662279 10.1104/pp.69.3.701 PMC426284

[ppl70597-bib-0024] Galmes, J. , J. M. Ochogavia , J. Gago , E. J. Roldan , J. Cifre , and M. A. Conesa . 2013. “Leaf Responses to Drought Stress in Mediterranean Accessions of *Solanum lycopersicum*: Anatomical Adaptations in Relation to Gas Exchange Parameters.” Plant, Cell & Environment 36: 920–935. 10.1111/pce.12022 23057729

[ppl70597-bib-0025] Galmes, J. , M. Ribas‐Carbo , H. Medrano , and J. Flexas . 2007. “Response of Leaf Respiration to Water Stress in Mediterranean Species With Different Growth Forms.” Journal of Arid Environments 68: 206–222.

[ppl70597-bib-0027] García‐Mata, C. , and L. Lamattina . 2009. “Nitric Oxide Induces Stomatal Closure and Enhances the Adaptive Plant Responses Against Drought Stress.” Plant Physiology 150: 531. 10.1104/pp.126.3.1196 PMC116475 11457969

[ppl70597-bib-0083] Gerszberg, A. , and K. Hnatuszko‐Konka . 2017. “Tomato Tolerance to Abiotic Stress: A Review of Most often Engineered Target Sequences.” Plant Growth Regulation 83, no. 2: 175–198.

[ppl70597-bib-0028] Girija, A. , F. J. Canales , B. S. Haddadi , et al. 2025. “Metabolomic Approaches Suggest Two Mechanisms of Drought Response Post‐Anthesis in Mediterranean Oat ( *Avena sativa* L.) Cultivars.” Physiologia Plantarum 177: e70181. 40148256 10.1111/ppl.70181 PMC11949858

[ppl70597-bib-0029] Golldack, D. , C. Li , H. Mohan , and N. Probst . 2014. “Tolerance to Drought and Salt Stress in Plants: Unraveling the Signaling Networks.” Frontiers in Plant Science 5: 151. 24795738 10.3389/fpls.2014.00151 PMC4001066

[ppl70597-bib-0030] Gupta, K. J. , A. R. Fernie , W. M. Kaiser , and J. T. van Dongen . 2011. “On the Origins of Nitric Oxide.” Trends in Plant Science 16: 160–168. 21185769 10.1016/j.tplants.2010.11.007

[ppl70597-bib-0031] Hammond, C. , M. M. Mira , B. T. Ayele , S. Renault , R. D. Hill , and C. Stasolla . 2020. “Over‐Expression of the *Zea mays* Phytoglobin (*ZmPgb1.1*) Alleviates the Effect of Water Stress Through Shoot‐Specific Mechanisms.” Plant Physiology and Biochemistry 155: 384–395. 32814275 10.1016/j.plaphy.2020.07.040

[ppl70597-bib-0032] Hartman, S. , Z. Liu , H. Van Veen , et al. 2019. “Ethylene‐Mediated Nitric Oxide Depletion Pre‐Adapts Plants to Hypoxia Stress.” Nature Communications 10: 4020. 10.1038/s41467-019-12045-4 PMC6728379 31488841

[ppl70597-bib-0034] Hebelstrup, K. H. , J. K. Shah , C. Simpson , et al. 2014. “An Assessment of the Biotechnological Use of Hemoglobin Modulation in Cereals.” Physiologia Plantarum 150: 593–603. 24118006 10.1111/ppl.12115

[ppl70597-bib-0035] Hebelstrup, K. H. , M. Van Zanten , J. Mandon , et al. 2012. “Haemoglobin Modulates NO Emission and Hyponasty Under Hypoxia‐Related Stress in *Arabidopsis thaliana* .” Journal of Experimental Botany 63: 5581–5591. 22915746 10.1093/jxb/ers210 PMC3444272

[ppl70597-bib-0033] Hebelstrup, K. H. , P. Hunt , E. Dennis , S. B. Jensen , and E. O. Jensen . 2006. “Hemoglobin Is Essential for Normal Growth of Arabidopsis Organs.” Physiologia Plantarum 127: 157–166.

[ppl70597-bib-0036] Hill, A. C. , and J. H. Bennett . 1970. “Inhibition of Apparent Photosynthesis by Nitrogen Oxides.” Atmospheric Environment 4: 341–348.

[ppl70597-bib-0037] Igamberdiev, A. U. , N. V. Bykova , and R. D. Hill . 2011. “Structural and Functional Properties of Class 1 Plant Hemoglobins.” IUBMB Life 63: 146–152. 21445844 10.1002/iub.439

[ppl70597-bib-0038] Ilyas, M. , M. Nisar , N. Khan , et al. 2021. “Drought Tolerance Strategies in Plants: a Mechanistic Approach.” Journal of Plant Growth Regulation 40: 926–944.

[ppl70597-bib-0039] Imran, M. , R. Shazad , S. Bilal , et al. 2021. “Exogenous Melatonin Mediates the Regulation of Endogenous Nitric Oxide in *Glycine max* L. to Reduce Effects of Drought Stress.” Environmental and Experimental Botany 188: 104511.

[ppl70597-bib-0040] Izanloo, A. , A. G. Condon , P. Langridge , M. Tester , and T. Schnurbusch . 2008. “Different Mechanisms of Adaptation to Cyclic Water Stress in Two South Australian Bread Wheat Cultivars.” Journal of Experimental Botany 59: 3327–3346. 18703496 10.1093/jxb/ern199 PMC2529232

[ppl70597-bib-0041] Jarosova, J. , and J. K. Kundu . 2010. “Validation of Reference Genes as Internal Control for Studying Viral Infections in Cereals by Quantitative Real‐Time RT‐PCR.” BMC Plant Biology 10: 146. 20630112 10.1186/1471-2229-10-146 PMC3095291

[ppl70597-bib-0042] Kamal, N. , N. Tsardakas Renhuldt , J. Bentzer , et al. 2022. “The Mosaic Oat Genome Gives Insights Into a Uniquely Healthy Cereal Crop.” Nature 606: 113–119. 35585233 10.1038/s41586-022-04732-y PMC9159951

[ppl70597-bib-0043] Khaipho‐Burch, M. , M. Cooper , J. Crossa , et al. 2023. “Genetic Modification Can Improve Crop Yields ‐ But Stop Overselling It.” Nature 621: 470–473. 37773222 10.1038/d41586-023-02895-w PMC11550184

[ppl70597-bib-0084] Krishna, R. , S. G. Karkute , W. A. Ansari , D. K. Jaiswal , J. P. Verma , and M. Singh . 2019. “Transgenic Tomatoes for Abiotic Stress Tolerance: Status and Way Ahead.” 3 Biotech 9, no. 4: 143. 10.1007/s13205-019-1665-0. PMC6423223 30944790

[ppl70597-bib-0044] Lau, S. E. , M. F. Hamdan , T. L. Pua , N. B. Saidi , and B. C. Tan . 2021. “Plant Nitric Oxide Signaling Under Drought Stress.” Plants 10: 360. 33668545 10.3390/plants10020360 PMC7917642

[ppl70597-bib-0045] Leshem, Y. Y. , and E. Haramaty . 1996. “The Characterization and Contrasting Effects of the Nitric Oxide Free Radical in Vegetative Stress and Senescence of *Pisum sativum* Linn Foliage.” Journal of Plant Physiology 148: 258–263.

[ppl70597-bib-0046] Lian, H. D. , C. Qin , J. Shen , and M. A. Ahanger . 2023. “Alleviation of Adverse Effects of Drought Stress on Growth and Nitrogen Metabolism in Mungbean (*Vigna radiata*) by Sulphur and Nitric Oxide Involves Up‐Regulation of Antioxidant and Osmolyte Metabolism and Gene Expression.” Plants 12: 3082. 37687329 10.3390/plants12173082 PMC10490269

[ppl70597-bib-0047] Livak, K. J. , and T. D. Schmittgen . 2001. “Analysis of Relative Gene Expression Data Using Real‐Time Quantitative PCR and the 2(T)(‐Delta Delta C) Method.” Methods 25: 402–408. 11846609 10.1006/meth.2001.1262

[ppl70597-bib-0048] Malmberg, R. L. , K. E. Smith , E. Bell , and M. L. Cellino . 1992. “Arginine Decarboxylase of Oats Is Clipped From a Precursor Into 2‐Polypeptides Found in the Soluble Enzyme.” Plant Physiology 100: 146–152. 16652937 10.1104/pp.100.1.146 PMC1075529

[ppl70597-bib-0049] Mishra, V. , P. Singh , D. K. Tripathi , F. J. Corpas , and V. P. Singh . 2021. “Nitric Oxide and Hydrogen Sulfide: An Indispensable Combination for Plant Functioning.” Trends in Plant Science 26: 1270–1285. 34417078 10.1016/j.tplants.2021.07.016

[ppl70597-bib-0051] Montilla‐Bascon, G. , D. Rubiales , K. H. Hebelstrup , et al. 2017. “Reduced Nitric Oxide Levels During Drought Stress Promote Drought Tolerance in Barley and Is Associated With Elevated Polyamine Biosynthesis.” Scientific Reports 7: 13311. 29042616 10.1038/s41598-017-13458-1 PMC5645388

[ppl70597-bib-0050] Montilla‐Bascon, G. , J. Mandon , F. J. M. Harren , L. A. J. Mur , S. M. Cristescu , and E. Prats . 2018. “Quantum Cascade Lasers‐Based Detection of Nitric Oxide.” In Nitric Oxide: Methods and Protocols, edited by A. Mengel and C. Lindermayr . Humana Press. 10.1007/978-1-4939-7695-9_5 29600450

[ppl70597-bib-0052] Montilla‐Bascon, G. , J. Sanchez‐Martin , N. Rispail , et al. 2013. “Genetic Diversity and Population Structure Among Oat Cultivars and Landraces.” Plant Molecular Biology Reporter 31: 1305–1314.

[ppl70597-bib-0053] Moreau, M. , C. Lindermayr , J. Durner , and D. F. Klessig . 2010. “NO Synthesis and Signaling in Plants ‐ Where Do We Stand?” Physiologia Plantarum 138: 372–383. 19912564 10.1111/j.1399-3054.2009.01308.x

[ppl70597-bib-0054] Mur, L. A. J. , A. Sivakumaran , J. Mandon , S. M. Cristescu , F. J. M. Harren , and K. H. Hebelstrup . 2012. “Haemoglobin Modulates Salicylate and Jasmonate/Ethylene‐Mediated Resistance Mechanisms Against Pathogens.” Journal of Experimental Botany 63: 4375–4387. 22641422 10.1093/jxb/ers116 PMC3421983

[ppl70597-bib-0055] Neill, S. J. , R. Desikan , A. Clarke , R. D. Hurst , and J. T. Hancock . 2002. “Hydrogen Peroxide and Nitric Oxide as Signalling Molecules in Plants.” Journal of Experimental Botany 53: 1237–1247. 11997372

[ppl70597-bib-0056] Peng, Y. , H. Yan , L. Guo , et al. 2022. “Reference Genome Assemblies Reveal the Origin and Evolution of Allohexaploid Oat.” Nature Genetics 54: 1248–1258. 35851189 10.1038/s41588-022-01127-7 PMC9355876

[ppl70597-bib-0057] PEPSICO . 2021. “*Avena sativa* – OT3098 v2.” https://wheat.pw.usda.gov/jb?data=/ggds/oat‐ot3098v2‐pepsico.

[ppl70597-bib-0058] Pilegaard, K. 2013. “Processes Regulating Nitric Oxide Emissions From Soils.” Philosophical Transactions of the Royal Society, B: Biological Sciences 27, no. 368: 20130126. 10.1098/rstb.2013.0126 PMC3682746 23713124

[ppl70597-bib-0059] Pissolato, M. D. , N. M. Silveira , P. J. C. Prataviera , et al. 2020. “Enhanced Nitric Oxide Synthesis Through Nitrate Supply Improves Drought Tolerance of Sugarcane Plants.” Frontiers in Plant Science 11: 970. 32695132 10.3389/fpls.2020.00970 PMC7339982

[ppl70597-bib-0060] Raeder, U. , and P. Broda . 1985. “Rapid Preparation of Dna From Filamentous Fungi.” Letters in Applied Microbiology 1: 17–20.

[ppl70597-bib-0061] Redmond, J. W. , and A. Tseng . 1979. “High‐Pressure Liquid‐Chromatographic Determination of Putrescine, Cadaverine, Spermidine and Spermine.” Journal of Chromatography 170: 479–481.

[ppl70597-bib-0062] Rezayian, M. , H. Ebrahimzadeh , and V. Niknam . 2023. “Metabolic and Physiological Changes Induced by Nitric Oxide and Its Impact on Drought Tolerance in Soybean.” Journal of Plant Growth Regulation 42: 1905–1918.

[ppl70597-bib-0063] Ribeiro, D. M. , R. Desikan , J. Bright , et al. 2009. “Differential Requirement for NO During ABA‐Induced Stomatal Closure in Turgid and Wilted Leaves.” Plant, Cell & Environment 32: 46–57. 10.1111/j.1365-3040.2008.01906.x 19021879

[ppl70597-bib-0064] Rigui, A. P. , V. Carvalho , A. L. W. Dos Santos , et al. 2019. “Fructan and Antioxidant Metabolisms in Plants of *Lolium perenne* Under Drought Are Modulated by Exogenous Nitric Oxide.” Plant Physiology and Biochemistry 145: 205–215. 31707248 10.1016/j.plaphy.2019.10.029

[ppl70597-bib-0065] Romero‐Puertas, M. C. , M. Perazzolli , E. D. Zago , and M. Delledonne . 2004. “Nitric Oxide Signalling Functions in Plant‐Pathogen Interactions.” Cellular Microbiology 6: 795–803. 15272861 10.1111/j.1462-5822.2004.00428.x

[ppl70597-bib-0066] Ruan, Q. , X. M. Bai , Y. Z. Wang , et al. 2024. “Regulation of Endogenous Hormone and miRNA in Leaves of Alfalfa (*Medicago sativa* L.) Seedlings Under Drought Stress by Endogenous Nitric Oxide.” BMC Genomics 25: 229. 38429670 10.1186/s12864-024-10024-8 PMC10908014

[ppl70597-bib-0067] Sahay, S. , E. Khan , and M. Gupta . 2019. “Nitric Oxide and Abscisic Acid Protects Against PEG‐Induced Drought Stress Differentially in *Brassica* Genotypes by Combining the Role of Stress Modulators, Markers and Antioxidants.” Nitric Oxide: Biology and Chemistry 89: 81–92. 31096008 10.1016/j.niox.2019.05.005

[ppl70597-bib-0072] Sanchez‐Martin, J. , D. Rubiales , F. Flores , et al. 2014. “Adaptation of Oat (*Avena sativa*) Cultivars to Autumn Sowings in Mediterranean Environments.” Field Crops Research 156: 111–122.

[ppl70597-bib-0073] Sanchez‐Martin, J. , D. Rubiales , J. C. Sillero , and E. Prats . 2012. “Identification and Characterization of Sources of Resistance in *Avena Sativa*, *A. byzantina* and *A. strigosa* Germplasm Against a Pathotype of *Puccinia coronata* f.sp. *Avenae* With Virulence Against the Pc94 Resistance Gene.” Plant Pathology 61: 315–322.

[ppl70597-bib-0068] Sánchez‐Martín, J. , F. J. Canales , J. K. S. Tweed , et al. 2018. “Fatty Acid Profile Changes During Gradual Soil Water Depletion in Oats Suggests a Role for Jasmonates in Coping With Drought.” Frontiers in Plant Science 9: 1077. 30131815 10.3389/fpls.2018.01077 PMC6090161

[ppl70597-bib-0069] Sanchez‐Martin, J. , J. Heald , A. KINGSTON‐Smith , et al. 2015. “A Metabolomic Study in Oats (*Avena sativa*) Highlights a Drought Tolerance Mechanism Based Upon Salicylate Signalling Pathways and the Modulation of Carbon, Antioxidant and Photo‐Oxidative Metabolism.” Plant, Cell & Environment 38: 1434–1452. 10.1111/pce.12501 25533379

[ppl70597-bib-0070] Sanchez‐Martin, J. , L. A. J. Mur , D. Rubiales , and E. Prats . 2012. “Targeting Sources of Drought Tolerance Within an *Avena* spp. Collection Through Multivariate Approaches.” Planta 236: 1529–1545. 22824964 10.1007/s00425-012-1709-8

[ppl70597-bib-0071] Sanchez‐Martin, J. , N. Rispail , F. Flores , et al. 2017. “Higher Rust Resistance and Similar Yield of Oat Landraces *Versus* Cultivars Under High Temperature and Drought.” Agronomy for Sustainable Development 37: 3.

[ppl70597-bib-0074] Shi, H. T. , T. T. Ye , J. K. Zhu , and Z. L. Chan . 2014. “Constitutive Production of Nitric Oxide Leads to Enhanced Drought Stress Resistance and Extensive Transcriptional Reprogramming in *Arabidopsis* .” Journal of Experimental Botany 65: 4119–4131. 24868034 10.1093/jxb/eru184 PMC4112625

[ppl70597-bib-0085] Shinwari, Z. K. , S. A. Jan , K. Nakashima , and K. Yamaguchi‐Shinozaki . 2020. “Genetic Engineering Approaches to Understanding Drought Tolerance in Plants.” Plant Biotechnology Reports 14, no. 2: 151–162.

[ppl70597-bib-0075] Singhal, R. K. , H. S. Jatav , T. Aftab , et al. 2021. “Roles of Nitric Oxide in Conferring Multiple Abiotic Stress Tolerance in Plants and Crosstalk With Other Plant Growth Regulators.” Journal of Plant Growth Regulation 40: 2303–2328.

[ppl70597-bib-0076] Slocum, R. D. , H. E. Flores , A. W. Galston , and L. H. Weinstein . 1989. “Improved Method for HPLC Analysis of Polyamines, Agmatine and Aromatic Monoamines in Plant Tissue.” Plant Physiology 89: 512–517. 11537449 10.1104/pp.89.2.512 PMC1055873

[ppl70597-bib-0077] Stevens, E. J. , K. W. Armstrong , H. J. Bezar , W. B. Griffin , and J. G. Hampton . 2004. “Fodder Oats: An Overview.” In Fodder Oats: A World Overview, edited by J. M. Suttie and S. G. Reynolds . Food and Agriculture Organization of the United Nations (FAO).

[ppl70597-bib-0078] Trull, M. C. , and R. L. Malmberg . 1994. “Puzzle Box, a Tobacco Line With Flowers That Mix Floral and Inflorescence Characteristics.” American Journal of Botany 81: 582–588.

[ppl70597-bib-0079] Ugalde, J. M. 2022. “Every Breath You Don't Take, I'll Be Helping You: Ethylene Promotes Hypoxia Tolerance.” Plant Physiology 190: 1085–1087. 35894688 10.1093/plphys/kiac347 PMC9516728

[ppl70597-bib-0080] Zangani, E. , S. Zehtab‐Salmasi , B. Andalibi , and A. A. Zamani . 2018. “Protective Effects of Nitric Oxide on Photosynthetic Stability and Performance of *Silybum marianum* Under Water Deficit Conditions.” Agronomy Journal 110: 555–564.

[ppl70597-bib-0081] Zhang, J. , M. J. Zhou , H. Zhou , et al. 2021. “Hydrogen Sulfide, a Signaling Molecule in Plant Stress Responses.” Journal of Integrative Plant Biology 63: 146–160. 33058490 10.1111/jipb.13022

[ppl70597-bib-0082] Zhao, Y. , X. H. Wei , Y. Long , and X. Z. Ji . 2020. “Transcriptional Analysis Reveals Sodium Nitroprusside Affects Alfalfa in Response to PEG‐Induced Osmotic Stress at Germination Stage.” Protoplasma 257: 1345–1358. 32556557 10.1007/s00709-020-01508-x

