# Supplementary material for: Phytoglobin Scavenging of Nitric Oxide Is Associated With Ethylene Reduction and Drought Tolerance in Oat (Avena sativa)
Source: Physiol Plant. 2025 Oct 26;177(6):e70597. doi: 10.1111/ppl.70597 (PMC12554845; doi:10.1111/ppl.70597)
Supplement: Supplementary file 1 — Data S1: Supporting Information. [file PPL-177-e70597-s001.pptx]

## Slide 1
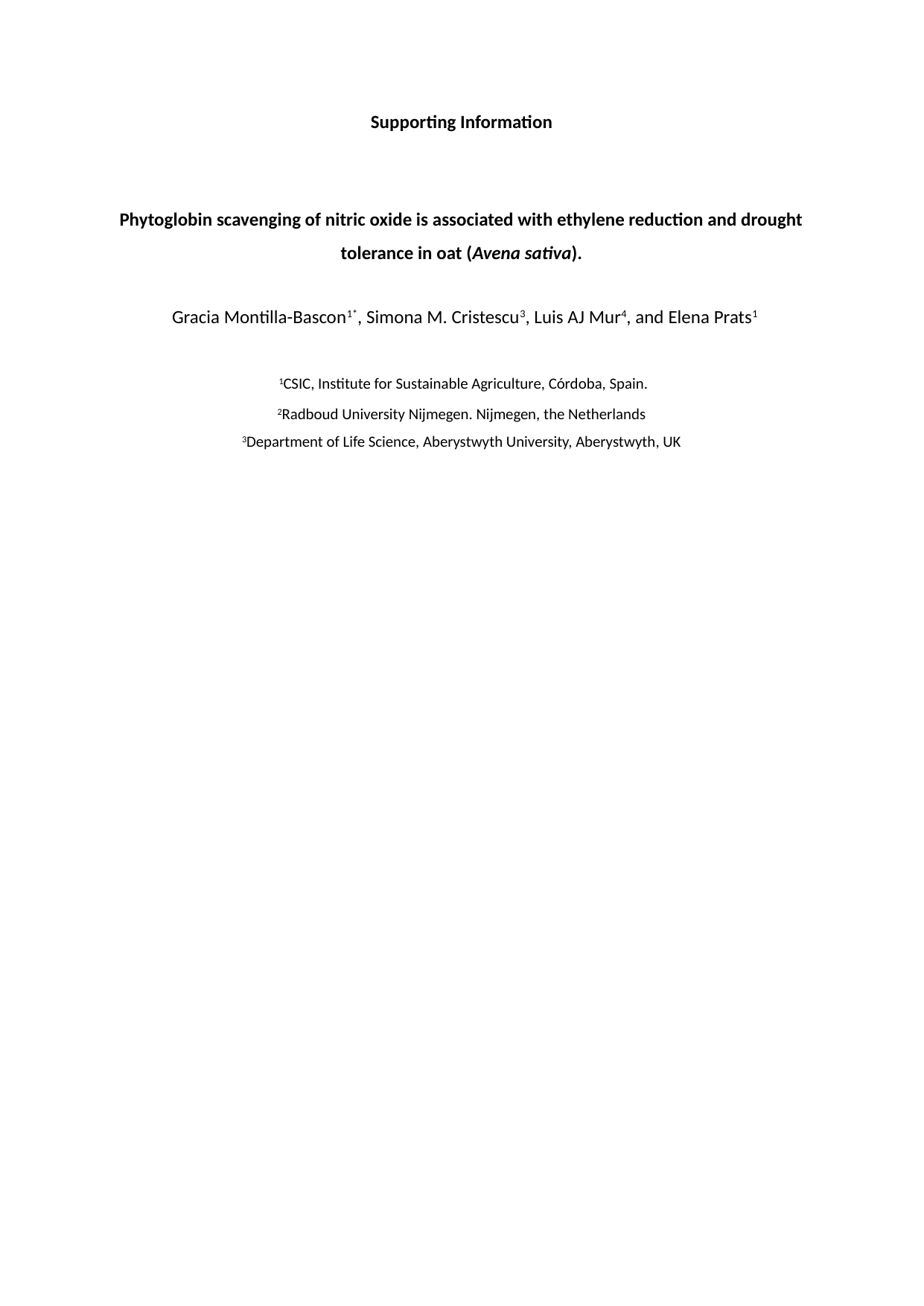

Supporting Information
Phytoglobin scavenging of nitric oxide is associated with ethylene reduction and drought tolerance in oat (Avena sativa).
 Gracia Montilla-Bascon1*, Simona M. Cristescu3, Luis AJ Mur4, and Elena Prats1
 1CSIC, Institute for Sustainable Agriculture, Córdoba, Spain.
2Radboud University Nijmegen. Nijmegen, the Netherlands
3Department of Life Science, Aberystwyth University, Aberystwyth, UK

## Slide 2
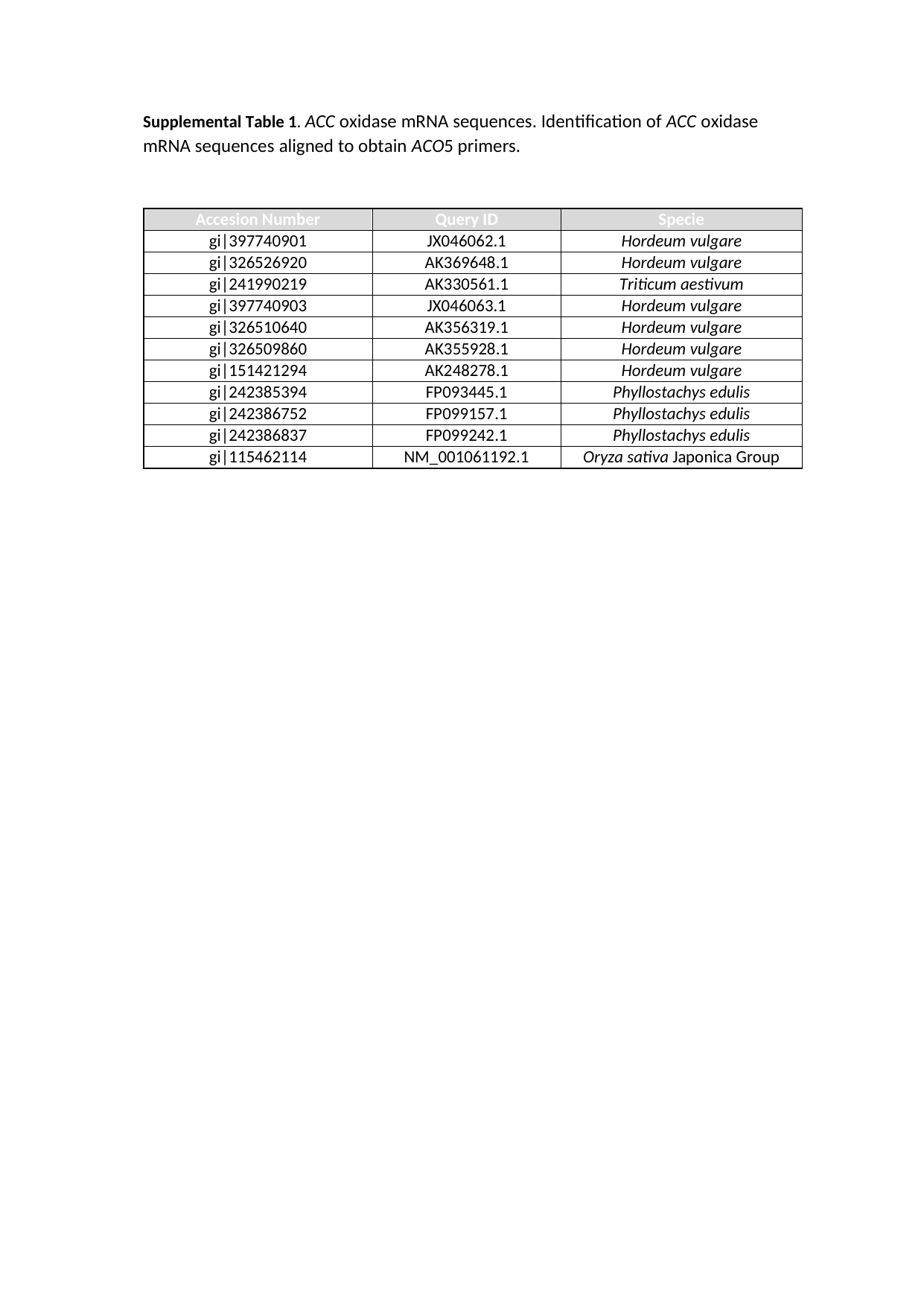

## Slide 3
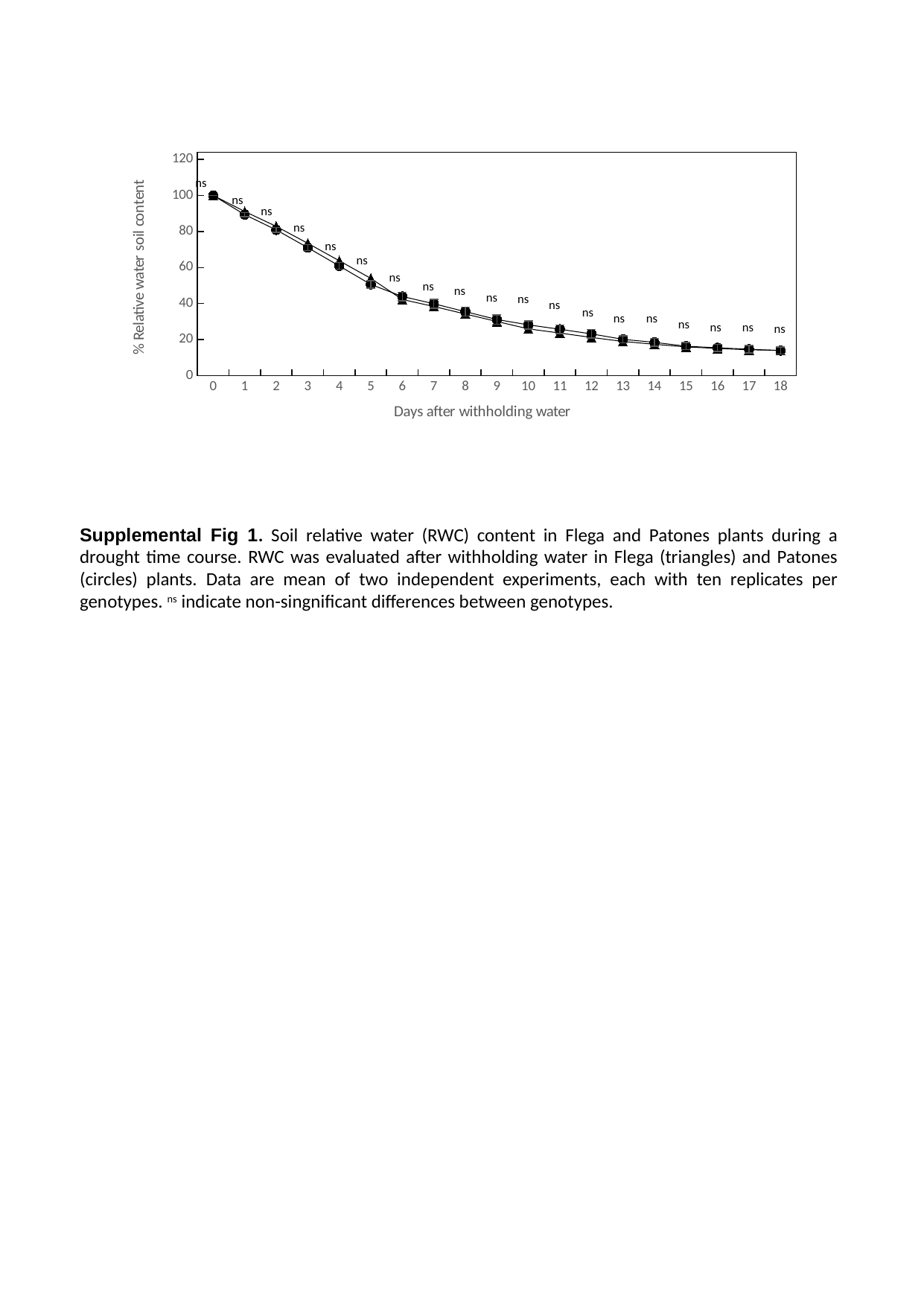

### Chart
| Category | Flega | Patones |
|---|---|---|
| 0 | 100.0 | 100.0 |
| 1 | 91.13790211500982 | 89.35911052681865 |
| 2 | 82.86877195281005 | 80.91454944247472 |
| 3 | 73.46408383372135 | 71.05180317106549 |
| 4 | 63.786136011414555 | 60.902488000729086 |
| 5 | 53.99432997943359 | 50.633769122506386 |
| 6 | 42.26792061512574 | 43.95895553824456 |
| 7 | 38.396741486203034 | 39.899229470109994 |
| 8 | 34.206759370192565 | 35.505173019893775 |
| 9 | 30.062320538051782 | 31.158878052832076 |
| 10 | 25.97935992375744 | 28.186247098692487 |
| 11 | 23.656652446403815 | 25.750411457811754 |
| 12 | 21.220086759375988 | 23.195172109044716 |
| 13 | 19.001377567743436 | 20.160325007504966 |
| 14 | 17.407362632304668 | 18.48867309709662 |
| 15 | 15.967039469134626 | 16.36096016198628 |
| 16 | 15.101717075610722 | 15.453491982050316 |
| 17 | 14.350252891761016 | 14.665427510000663 |
| 18 | 14.072611057467356 | 13.902321148271783 |ns
ns
ns
ns
ns
ns
ns
ns
ns
ns
ns
ns
ns
ns
ns
ns
ns
ns
ns
Supplemental Fig 1. Soil relative water (RWC) content in Flega and Patones plants during a drought time course. RWC was evaluated after withholding water in Flega (triangles) and Patones (circles) plants. Data are mean of two independent experiments, each with ten replicates per genotypes. ns indicate non-singnificant differences between genotypes.

## Slide 4
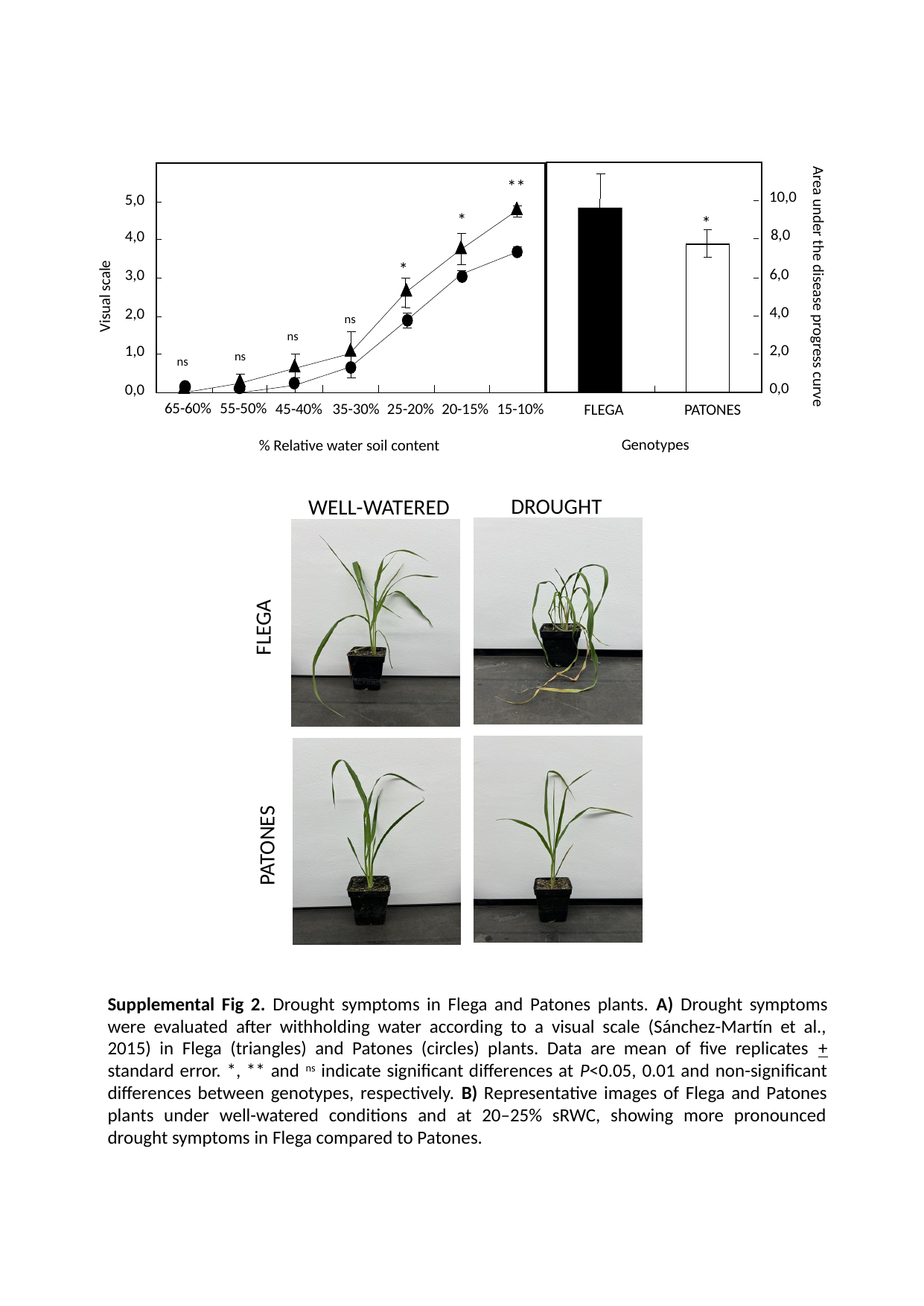

5,0
4,0
3,0
Area under the disease progress curve
Visual scale
2,0
1,0
0,0
65-60%
55-50%
45-40%
35-30%
25-20%
20-15%
15-10%
% Relative water soil content
10,0
8,0
6,0
4,0
2,0
0,0
FLEGA
PATONES
**
*
*
*
ns
ns
ns
ns
Genotypes
DROUGHT
WELL-WATERED
FLEGA
PATONES
Supplemental Fig 2. Drought symptoms in Flega and Patones plants. A) Drought symptoms were evaluated after withholding water according to a visual scale (Sánchez-Martín et al., 2015) in Flega (triangles) and Patones (circles) plants. Data are mean of five replicates + standard error. *, ** and ns indicate significant differences at P<0.05, 0.01 and non-significant differences between genotypes, respectively. B) Representative images of Flega and Patones plants under well-watered conditions and at 20–25% sRWC, showing more pronounced drought symptoms in Flega compared to Patones.
